# A Clinical Prediction Rule for Histological Chorioamnionitis in Preterm Newborns

**DOI:** 10.1371/journal.pone.0046217

**Published:** 2012-10-05

**Authors:** Jasper V. Been, Sizzle F. Vanterpool, Jasmijn D. E. de Rooij, G. Ingrid J. G. Rours, René F. Kornelisse, Martien C. J. M. van Dongen, Christel J. A. W. van Gool, Ronald R. de Krijger, Peter Andriessen, Luc J. I. Zimmermann, Boris W. Kramer

**Affiliations:** 1 Department of Paediatrics, Maastricht University Medical Centre, Maastricht, The Netherlands; 2 School for Public Health and Primary Care (CAPHRI), Maastricht University Medical Centre, Maastricht, The Netherlands; 3 School for Oncology and Developmental Biology (GROW), Maastricht University Medical Centre, Maastricht, The Netherlands; 4 Department of Paediatrics, Máxima Medical Centre, Veldhoven, The Netherlands; 5 Department of Paediatrics, Erasmus MC – Sophia Children's Hospital, Rotterdam, The Netherlands; 6 Department of Epidemiology, Maastricht University Medical Centre, Maastricht, The Netherlands; 7 Department of Pathology, Erasmus MC – University Medical Centre, Rotterdam, The Netherlands; Hôpital Robert Debré, France

## Abstract

**Background:**

Histological chorioamnionitis (HC) is an intrauterine inflammatory process highly associated with preterm birth and adverse neonatal outcome. HC is often clinically silent and diagnosed postnatally by placental histology. Earlier identification could facilitate treatment individualisation to improve outcome in preterm newborns.

**Aim:**

Develop a clinical prediction rule at birth for HC and HC with fetal involvement (HCF) in preterm newborns.

**Methods:**

Clinical data and placental pathology were obtained from singleton preterm newborns (gestational age ≤32.0 weeks) born at Erasmus UMC Rotterdam from 2001 to 2003 (derivation cohort; n = 216) or Máxima MC Veldhoven from 2009 to 2010 (validation cohort; n = 206). HC and HCF prediction rules were developed with preference for high sensitivity using clinical variables available at birth.

**Results:**

HC and HCF were present in 39% and 24% in the derivation cohort and in 44% and 22% in the validation cohort, respectively. HC was predicted with 87% accuracy, yielding an area under ROC curve of 0.95 (95%CI = 0.92–0.98), a positive predictive value of 80% (95%CI = 74–84%), and a negative predictive value of 93% (95%CI = 88–96%). Corresponding figures for HCF were: accuracy 83%, area under ROC curve 0.92 (95%CI = 0.88–0.96), positive predictive value 59% (95%CI = 52–62%), and negative predictive value 97% (95%CI = 93–99%). External validation expectedly resulted in some loss of test performance, preferentially affecting positive predictive rather than negative predictive values.

**Conclusion:**

Using a clinical prediction rule composed of clinical variables available at birth, HC and HCF could be predicted with good test characteristics in preterm newborns. Further studies should evaluate the clinical value of these rules to guide early treatment individualisation.

## Introduction

Chorioamnionitis is an antenatal inflammatory state of the intrauterine environment strongly associated with prematurity [1]. Around 40% of infants born before 32 weeks gestation have been exposed to chorioamnionitis [1]–[3], which often is a clinically silent process. Exposure to chorioamnionitis is known to affect several organ systems in the fetus [4]. Its presence in placentas from preterm infants has been associated with decreased respiratory distress syndrome, but increased incidences of bronchopulmonary dysplasia (BPD), necrotising enterocolitis (NEC) and neurologic sequelae, including white matter damage and cerebral palsy [2], [4]–[6]. These effects have generally been shown to be more pronounced when additional signs of fetal inflammation including funisitis are present [2], [4], [5].

Recent studies have suggested differential effects of distinct treatments in infants with varying degrees of chorioamnionitis as opposed to non-exposed infants in the early neonatal period [7]–[10]. Thus, perinatal identification of chorioamnionitis-exposed infants could facilitate the development of subgroup-targeted early intervention strategies to improve outcome in this group. Evidence from randomised controlled trials and observational studies suggests that chorioamnionitis-exposed infants may distinctively benefit from increased surfactant dosing [8], [10], restrictive use of invasive and prolonged ventilation [8], [11]–[13], as well as postnatal corticosteroid administration to improve BPD-free survival [9]. The gold standard for diagnosing chorioamnionitis is placental histological examination [14]. Unfortunately in current practice, the final results of placental pathology may take days if not weeks, hindering its use as an indicator to guide early postnatal therapy. To overcome this problem, the ability of biological markers to detect chorioamnionitis before or shortly after birth has been investigated. Indeed, several markers and marker patterns in amniotic fluid and umbilical cord blood have been shown to carry predictive value for histological chorioamnionitis [15]–[17]. However, to date their use is experimental, and sensitivity and specificity have generally shown to be at best moderate.

Several clinical variables are well known to be distributed in a differential pattern among preterm infants with and without chorioamnionitis [2], [5], [18]. In the current study we aimed to develop a clinical prediction rule for both histological chorioamnionitis (HC) and chorioamnionitis with fetal involvement (HCF) at birth in preterm infants, composed solely of clinical variables available at that time.

## Methods

### Ethics statement

The derivation cohort was part of a study approved by the Medical Ethics Committee for Research on Human Subjects of the Erasmus University MC. Written parental consent was obtained. According to Dutch law a waiver for ethical assessment and parental consent was provided by the local Medical Ethical Committee of the Máxima Medical Centre for development and use of the validation cohort, considering that retrospective and anonymised data collection was performed using routinely collected medical chart data solely.

### Derivation cohort

The clinical prediction rule was developed in a prospective cohort described previously [2], [8], [19]. Pregnant women, who delivered between May 2001 and February 2003 in the Erasmus University MC–Sophia Children's Hospital in Rotterdam, Netherlands, at a gestational age of 32.0 weeks or less, were eligible for the study. Newborns were enrolled immediately after delivery when admitted to the neonatal intensive care unit (NICU; level III). Trained research nurses unaware of results of placental histology prospectively collected relevant clinical data. Multiple births were excluded from analysis, as were newborns with severe congenital abnormalities.

Placentas and membranes were fixed in formalin directly after delivery. Sampling was done according to a standard protocol and included at least two membrane rolls, two cross-sections of the cord, and three representative blocks of the placental disk. Tissues were embedded in paraffin until examination. A single pathologist (RRdK) examined all placentas in a blinded fashion for presence of chorioamnionitis and additional fetal inflammatory response, according to the Amniotic Fluid Infection Nosology Committee guidelines [14]. Accordingly, fetal involvement included any of the following: chorionic vasculitis, umbilical phlebitis or vasculitis, (subacute) necrotising funisitis, or concentric umbilical perivasculitis.

### Predictors

Clinical variables were considered as potential predictors when they were deemed to be readily available in daily practice, were available in the prospectively collected database of the derivation cohort, and were potentially associated with HC and/or HCF – either positively or negatively – or had been associated with these entities in prior clinical studies. According to these criteria, the following clinical parameters were evaluated as potential predictors of HC and HCF: ethnicity (self-classified); maternal age; gravidity; parity; antenatal steroid administration (betamethasone 12 mg intramuscularly, repeated after 24 hours); preeclampsia (new onset hypertension [blood pressure >140/90 mm Hg or mean arterial pressure >105 mm Hg] with proteinuria); HELLP syndrome (clinical presentation of intravascular haemolysis, elevated liver enzymes, and a low platelet count); preterm premature rupture of membranes (PPROM); clinical chorioamnionitis (maternal temperature >38.0°C with no other focus, and two or more of the following: uterine tenderness, malodorous vaginal discharge, maternal leukocytosis [WBC>15,000 cells/µL], raised serum C-reactive protein [CRP>15 mg/L], maternal tachycardia [>100 bpm], and fetal tachycardia [>160 bpm]); mode of delivery; gestational age (preferably estimated by ultrasonography or otherwise by using the last menstrual period when reliable); gender; birth weight; being small for gestational age (SGA; birth weight <10^th^ percentile of the gender-specific mean for gestational age); and placental weight.

### Clinical prediction rule development

Clinical prediction rule development methodology was guided by published standards [20]. Univariable analyses were performed to identify relevant clinical variables that differed between the groups (‘no HC’ versus ‘HC’, and ‘no HCF’ versus ‘HCF’), using χ^2^-test, Student *t* test, or Mann-Whitney U-test where appropriate (alpha <0.10). These were entered into a backward logistic regression model to predict either HC or HCF. Before entry, continuous variables were dichotomised based on the most discriminative cut-off value on the ROC curve. The final model was selected using the likelihood ratio method with an alpha level of 0.10. Next, for each predictor in the model the beta value was divided by the lowest beta value in the model and rounded off to the nearest integer. A weighted score was thus obtained for each predictor and used to develop a clinical prediction rule for HC or HCF. ROC curves were computed based on individual composite scores calculated for each patient to determine the optimum cut-off value for prediction, defined as the cut-off with a sensitivity of at least 0.80 and maximum specificity. Relevant test statistics were computed from two-by-two contingency tables. Internal model performance was estimated using leave-one-out cross-validation.

### Validation cohort

The clinical prediction rule was externally validated in a cohort of inborn singleton newborns with a gestational age ≤32.0 weeks admitted to the NICU (level III) of the Máxima Medical Centre, Veldhoven, Netherlands between January 1, 2009 and December 31, 2010. Retrospective data retrieval from maternal and neonatal medical charts was performed for the variables included in both clinical prediction rules. Data were anonymised before storage. Similar to the derivation cohort, placenta sampling and fixation was standardised and presence of chorioamnionitis and additional fetal inflammatory response was scored according to international guidelines [14]. Individual composite scores were calculated for both prediction rules with positive test scores defined by the optimum cut-off values identified in the derivation cohort. Again, relevant test statistics were computed from two-by-two contingency tables. Analyses were performed using SPSS 16.0 software (SPSS, Inc, Chicago, IL).

## Results

### Clinical prediction rule development

The derivation cohort was composed of all 216 singletons from a cohort of 301 newborns described previously [2], [8], [19]. Of these, 84 (39%) had HC, while 51 (24%) had additional signs of fetal involvement. Placental pathology and relevant clinical data were available for all mother-newborn pairs. Newborns with HC were more often born vaginally, were of lower gestational age but had a higher birth weight and placental weight as compared to those without HC (Table 1). Accordingly, they were less likely to be SGA. Their mothers more often experienced PPROM and clinical chorioamnionitis, and less often had preeclampsia and HELLP syndrome. The same differences were present between newborns with HCF and those without, with the exception of birth weight, which was not significantly different between these groups.

**Table 1:** Derivation cohort baseline characteristics.

	*No HC (n = 132)*	*HC (n = 84)*	*P-value*	*No HCF (n = 165)*	*HCF (n = 51)*	*P-value*
Ethnicity			0.38			0.38
Western Europe	92 (70)	57 (68)		118 (72)	31 (61)	
Eastern Europe/Asia	3 (2)	5 (6)		6 (4)	2 (4)	
Mediterranean	11 (8)	7 (8)		12 (7)	6 (12)	
Sub-Saharan Africa	11 (8)	10 (12)		13 (8)	8 (16)	
South America/Caribbean	15 (11)	5 (6)		16 (10)	4 (8)	
Maternal age (years; mean ± SD)	30.5±5.7	30.3±5.6	0.75	30.4±5.6	30.4±5.8	0.95
Gravidity (median (IQR))	1 (1–3)	2 (1–3)	0.17	1 (1–2)	2 (1–3)	0.09
Parity (median (IQR))	1 (1–2)	1 (1–2)	0.45	1 (1–2)	1 (1–2)	0.29
Betamethasone (% full course)	92 (70)	65 (77)	0.22	120 (73)	37 (73)	0.98
Preeclampsia (%)	88 (67)	3 (4)	<.001	90 (55)	1 (2)	<.001
HELLP (%)	58 (44)	3 (4)	<.001	61 (37)	0 (0)	<.001
PPROM (%)	11 (8)	49 (58)	<.001	24 (15)	36 (71)	<.001
Clinical chorioamnionitis (%)	10 (8)	52 (62)	<.001	25 (15)	37 (73)	<.001
Mode of delivery (% vaginal)	17 (13)	59 (70)	<.001	40 (24)	36 (71)	<.001
Gestational age (weeks; mean ± SD)	29.7±1.6	28.6±2.1	<.001	29.6±1.6	28.2±2.2	<.001
Gender (% male)	63 (48)	49 (58)	0.13	86 (52)	26 (51)	0.89
Birth weight (grams; mean ± SD)	1069±329	1240±370	<.001	1126±353	1166±362	0.50
Small for gestational age (%)	63 (48)	3 (4)	<.001	64 (39)	2 (4)	<.001
Placental weight (g; mean ± SD)	225±75	297±101	<.001	243±93	286±87	0.004

Derivation cohort baseline characteristics. for mother-infant pairs with and without histological chorioamnionitis (HC; left), and with and without histological chorioamnionitis with fetal involvement (HCF; right). Abbreviations: SD = standard deviation; IQR = interquartile range; HELLP = haemolysis, elevated liver enzymes, low platelets; PPROM = preterm premature rupture of membranes.

**Figure 1:**
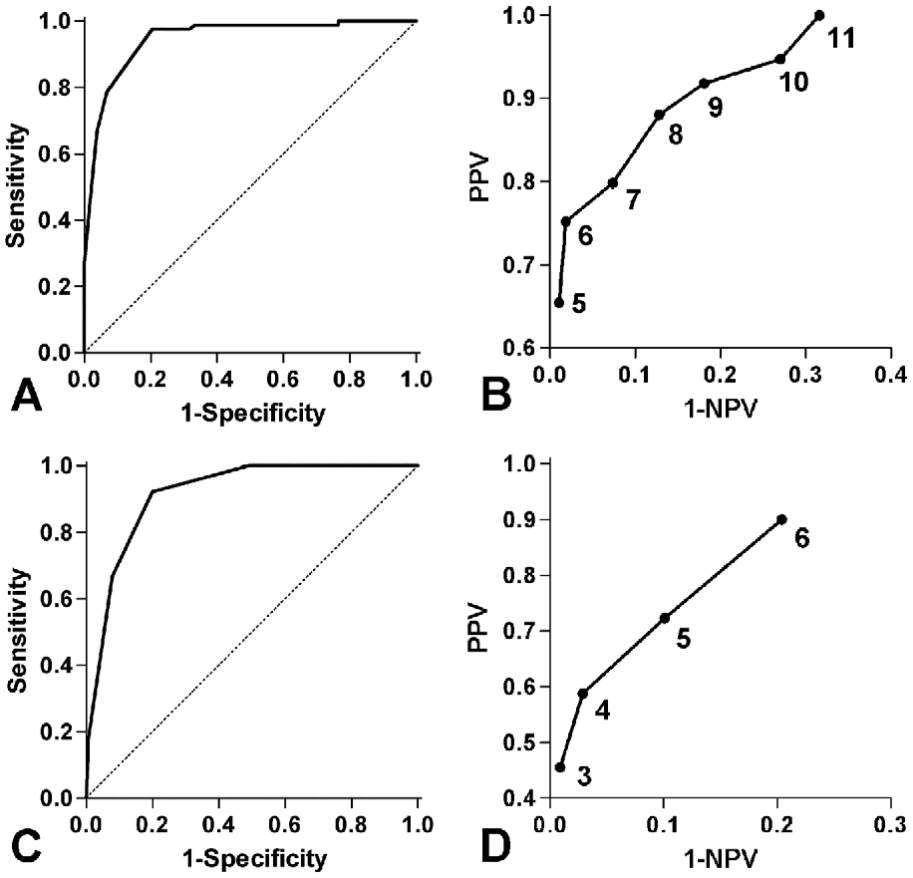
ROC curve and PPV-NPV plot of clinical prediction rule for histological chorioamnionitis (A+B) and histological chorioamnionitis with fetal involvement (C+D). Figures in PPV-NPV plots indicate inclusive (≥) cut-off values for positive test scores.

The final logistic regression models for prediction of HC and HCF are shown in Tables 2 and 3. Clinical prediction rules based on the weighted beta values were computed, yielding the following formulas: HC score = 3×‘no preeclampsia’+3×‘not SGA’+2×‘clinical chorioamnionitis’+2×‘gestational age ≤28.0 weeks’+1×‘PPROM’+1×‘vaginal delivery’ (maximum score = 12; Table 2), and HCF score = 2×‘no preeclampsia’+1×‘PPROM’+1×‘gestational age ≤28.0 weeks’+1×‘not SGA’+1×‘clinical chorioamnionitis’ (maximum score = 6; Table 3). ROC curves showed high predictive ability for both clinical prediction rules, with an area under the curve of 0.95 (95% CI 0.92–0.98) for HC and 0.92 (95% CI 0.88–0.96) for HCF (Figure 1). These values were very similar to those derived from prediction by the actual regression model itself (0.95 [95% CI 0.93–0.98], and 0.93 [95% CI 0.89–0.96], respectively), showing no important loss of information by simplification into a points-based clinical prediction rule. Based on a preference for high sensitivity over specificity, final cut-off values of ≥7 and ≥4 were selected for HC and HCF, respectively. Using these cut-offs, positive and negative predictive values of the HC clinical prediction rule were 80% (95% CI 74–84%) and 93% (95% CI 88–96%), respectively (Figure 1). Corresponding figures for the HCF clinical prediction rule were 59% (95% CI 52–62%) and 97% (95% CI 93–99%). Internal cross-validation yielded 87% accuracy for the HC clinical prediction rule and of 83% for the HCF prediction rule. Additional test characteristics are shown in Table 4.

**Table 2:** Logistic regression model and clinical prediction rule for histological chorioamnionitis.

*Factor*	*Beta*	*SE*	*P-value*	*Prediction rule (points per item)*
No preeclampsia	2.72	0.74	<.001	3
Not SGA	2.50	0.84	.003	3
Clinical chorioamnionitis	1.64	0.55	.003	2
Gestational age ≤28.0 wks	1.34	0.61	.028	2
PPROM	1.02	0.52	.048	1
Vaginal delivery	0.87	0.51	.086	1
Intercept	−6.89	1.12	<.001	Total = 12

Logistic regression model and clinical prediction rule for prediction of histological chorioamnionitis. Abbreviations: SE = standard error; SGA = small for gestational age; PPROM = preterm premature rupture of membranes.

**Table 3:** Logistic regression model and clinical prediction rule for histological chorioamnionitis with fetal involvement.

*Factor*	*Beta*	*SE*	*P-value*	*Prediction rule (points per item)*
No preeclampsia	2.26	1.10	.039	2
PPROM	1.72	0.49	<.001	1
Gestational age ≤27.0 wks	1.70	0.59	.004	1
Not SGA	1.60	0.91	.077	1
Clinical chorioamnionitis	1.30	0.47	.006	1
Intercept	−6.07	1.28	<.001	Total = 6

Logistic regression models and clinical prediction rules for prediction of histologic chorioamnionitis with fetal involvement. Abbreviations: SE = standard error; PPROM = preterm premature rupture of membranes; SGA = small for gestational age.

**Table 4:** Clinical prediction rule test characteristics.

	*HC prediction rule*		*HCF prediction rule*	
	*Derivation cohort*	*Validation cohort*	*Derivation cohort*	*Validation cohort*
*Characteristic*	*Estimate (95% CI)*	*Estimate (95% CI)*	*Estimate (95% CI)*	*Estimate (95% CI)*
Area under ROC curve	0.95 (0.92–0.98)	0.81 (0.74–0.87)	0.92 (0.88–0.96)	0.83 (0.77–0.89)
Positive test score	≥7		≥4	
Sensitivity	0.89 (0.82–0.94)	0.89 (0.80–0.94)	0.92 (0.82–0.97)	0.80 (0.66–0.90)
Specificity	0.86 (0.81–0.89)	0.64 (0.58–0.67)	0.80 (0.77–0.82)	0.75 (0.71–0.78)
Accuracy (%)	87 (82–91)	73 (67–78)	83 (78–85)	76 (70–80)
PPV (%)	80 (74–84)	60 (54–64)	59 (52–62)	47 (39–53)
NPV (%)	93 (88–96)	90 (83–95)	97 (93–99)	93 (88–97)
LLR+	6.20 (4.36–8.36)	2.44 (1.92–2.88)	4.61 (3.52–5.30)	3.18 (2.26–4.02)
LLR−	0.13 (0.07–0.22)	0.18 (0.08–0.34)	0.10 (0.03–0.24)	0.27 (0.13–0.48)
Diagnostic Odds Ratio	49.6 (19.9–127.6)	13.6 (5.6–34.3)	47.0 (14.8–166.2)	11.9 (4.7–31.0)

Clinical prediction rule test characteristics for histological chorioamnionitis (HC) and histological chorioamnionitis with fetal involvement (HCF). Abbreviations: ROC = receiver operating characteristics; PPV = positive predictive value; NPV = negative predictive value; LLR+ = likelihood ratio of positive test; LLR− = likelihood ratio of negative test.

### External validation

The validation cohort was derived from a birth cohort of 206 inborn singleton newborns. Placental pathology was available for 183 (89%) of these. Distribution of the predictor variables over both cohorts is shown in Table 5. Newborns with missing placental pathology were less often delivered by caesarean section (17 vs. 45%, p = 0.01). No other important differences in predictor variables were present between newborns with and without available placental pathology. HC was diagnosed in 80 placentas (44%), and HCF was present in 40 (22%). Validation of the clinical prediction rules led to an expected small drop in accuracy (73% for HC and 76% for HCF), affecting PPV (60 [95% CI 54–64] and 47 [39–53], respectively) rather than NPV (90 [83–95] and 93 [88–97], respectively).

**Table 5:** Distribution of predictor variables among different subsets within the derivation and validation cohorts.

	*Derivation cohort* *(n = 216)*				*Validation cohort* *(n = 206)*					
	*No HC* *(n = 132)*	*HC* *(n = 84)*	*No HCF* *(n = 165)*	*HCF* *(n = 51)*	*Placental pathology unavailable* *(n = 23)*	*Placental pathology available* *(n = 183)*	*Placental pathology available* *(n = 183)*			
							*No HC* *(n = 103)*	*HC* *(n = 80)*	*No HCF* *(n = 143)*	*HCF* *(n = 40)*
No preeclampsia (%)	44 (33)	81 (96)	75 (46)	50 (98)	19 (83)	135 (74)	60 (58)	75 (94)	95 (66)	40 (100)
Not SGA (%)	69 (52)	81 (96)	101 (61)	49 (96)	22 (96)	162 (89)	83 (81)	78 (98)	122 (85)	40 (100)
Clinical chorioamnionitis (%)	10 (8)	52 (62)	25 (15)	37 (73)	0 (0)	13 (7)	0 (0)	13 (16)	4 (3)	9 (23)
Gestational age ≤27.0 wks (%)			16 (10)	20 (39)	3 (13)	24 (13)			16 (11)	8 (20)
Gestational age ≤28.0 wks (%)	26 (20)	33 (39)			3 (13)	41 (22)	19 (18)	22 (28)		
PPROM (%)	11 (8)	49 (58)	24 (15)	36 (71)	10 (44)	62 (34)	20 (19)	42 (53)	33 (23)	29 (73)
Vaginal delivery (%)	17 (13)	59 (70)	40 (24)	36 (71)	19 (83)	101 (55)	37 (36)	64 (80)	67 (47)	34 (85)

Abbreviations: HC = histological chorioamnionitis; HCF = histological chorioamnionitis with fetal involvement.

## Discussion

In a well-defined cohort of very preterm newborns reported previously [2], [8], [19], we developed a clinical prediction rule for histological chorioamnionitis and for histological chorioamnionitis with fetal involvement. Using a simple set of clinical variables generally available in the clinical setting, both HC and HCF could be predicted at birth with high accuracy, with an expected small drop in prediction rule performance at external validation. The prediction rules presented here carry future potential in facilitation of subgroup-targeted early intervention strategies.

The current study has several strengths. Development and validation were performed according to accepted standards [20], [21]. A large, prospective cohort with complete data was used for prediction rule development. Predictors were well defined and outcome assessment was performed in a blinded fashion according to widely used published standards [14]. External validation was performed using a cohort of consecutively inborn singletons with a high rate of available placental pathology. The temporal and geographical spacing between the derivation and validation cohort increase generalisability of the results. Conversely, external validation is limited somewhat by the retrospective nature of the validation cohort. Furthermore the higher rate of vaginal deliveries in newborns without placental pathology indicates a minor selection bias, the effect of which is likely to be small given the low weight carried by mode of delivery in the prediction rules. It is important to note that some diagnostic performance was lost at external validation, a phenomenon observed almost invariably during any process of clinical prediction rule development [22]. During further evaluation of the decision rule it must be kept in mind that it is derived from a cohort of very preterm singleton newborns, and should therefore not be extrapolated to multiplets and more mature newborns without further testing. Application of our model to multiplets in the derivation cohort showed it had no discriminative value in these newborns (not shown), which is in agreement with the distinct mechanisms underlying preterm delivery in this subgroup. Finally, because several diagnostic criteria for histological chorioamnionitis exist [14], [23]–[26], the generalisability of our prediction rule for chorioamnionitis diagnosed by criteria other than those by Redline and co-workers [14], requires evaluation.

To date, chorioamnionitis remains a troublesome diagnostic entity. Most cases are clinically silent and there is no reliable way of determining its presence, let alone severity, in a non-invasive manner before delivery [27], [28]. Results of placental pathology, the current gold standard for diagnosis, often take days to weeks to obtain. The prediction rules presented here underline the distinct clinical characteristics of affected patients as compared to other preterm newborns. As noted earlier, the primary causes of preterm birth among singletons – preeclampsia and chorioamnionitis – rarely co-exist [3], [29]. Therefore it is not surprising to see how factors known to characterise either of these groups – preeclampsia and SGA on the one hand, clinical chorioamnionitis, PPROM and vaginal delivery on the other – comprise the major part of the prediction models. The additional contribution of gestational age is explained by its known inverse relationship with chorioamnionitis incidence: lower gestational age increases the likelihood of chorioamnionitis [1], [3]. The mutual exclusion of many of the aforementioned variables would potentially favour classification and regression-tree (CART) analyses rather than points-based prediction rules as the most distinctive way to identify chorioamnionitis-exposed newborns. CART analyses were evaluated simultaneously in the current study and were disregarded for their slightly worse test performance (data not shown). Similarly, it may seem appealing to restrict the analyses to subgroups of patients particularly at risk of histological chorioamnionitis, such as those with preterm labour or PPROM. However, PPROM for example was absent in 42% of newborns in the derivation cohort and in 47% in the validation cohort. Consequently, restriction of analyses to PPROM patients would not have allowed identifying over 80% of patients with histological chorioamnionitis in the cohort, and thus using a combination of risk factors in a model-based approach as presented here is favourable.

Few previous studies have evaluated multivariable models for chorioamnionitis prediction. Using a combination of clinical and laboratory or ultrasound parameters, others were able to predict intra-amniotic inflammation and infection, but with test characteristics inferior to those presented here [30]–[32]. Balaguer and colleagues showed that an algorithm could improve classification of the alleged underlying causes of prematurity in a small cohort [33]. However, it is unclear whether placental histology or other tests were performed to confirm chorioamnionitis. Several biological markers have previously been evaluated as a diagnostic tool to identify the presence and severity of chorioamnionitis shortly after birth. The marker most extensively investigated in this regard is probably interleukin-6 (IL-6), predominantly measured in cord blood. Its sensitivity and specificity for diagnosing HC range from 73% to 74%, and 77% to 85%, respectively [15], [34], [35]. Diagnostic properties reported for HCF are within the same range [16], [17], [34], [35]. Several other molecular and haematologic markers have been evaluated, but to date none has shown to carry sufficient predictive ability to justify routine clinical use [17], [34], [36], [37]. Recent studies showed that test accuracy can be enhanced by combining several molecular markers in amniotic or cervical fluid [38], [39]. However, external validation was not performed, and obvious drawbacks remain the need for an invasive procedure (for amniotic fluid), as well as costs and time required for proteomic profiling.

To the best of our knowledge, the diagnostic properties of the models presented here are at least equivalent to those of any non-invasive biological marker reported to date, most of which have not been externally validated. Moreover, the prediction rules are easy to use, cheap, and their results are readily available. The models may be used to identify at birth newborns exposed to various degrees of antenatal inflammation. As such, they can facilitate the development of decision rules in future studies to optimise and individualise treatment of preterm newborns. In addition they may be used for predefined subgroup analyses in clinical trials to investigate their value in predicting differential therapeutic responses. Negative predictive value were particularly well preserved during external validation of the prediction rules presented here, making these predominantly useful to guide targeted therapies with large subgroup-associated benefits and little adverse effects at the population level. Whereas in the current study we have preferred high sensitivity to specificity the model obviously allows for tailored selection of a less conservative cut-off value to increase specificity over sensitivity.

Evidence from observational studies suggests that infants exposed to varying degrees of antenatal inflammation may particularly benefit from increased surfactant dosing [8], [10] and restrictive use of invasive ventilation [8], [11]–[13]. Furthermore, a randomised controlled trial identified subgroup-associated benefits of postnatal corticosteroids in infants with chorioamnionitis [9]. Results of placental pathology are generally unavailable when decisions regarding the initiation of such interventions are required. Future studies should evaluate the potential value of the use of the clinical prediction rules presented here to guide such decision-making. Another potential field of interest is antibiotic prophylaxis for prevention of early onset sepsis in preterm newborns. Its use is widespread, although serious potential side-effects are well recognised [40]–[42]. Individualised antibiotic prophylaxis based on prediction of sepsis risk may well improve outcome at the group level by reducing unnecessary and potentially harmful use of antibiotics. The clinical prediction rules presented here could guide such individualisation of therapy.

## Conclusion

Development of prediction strategies to identify subgroups of preterm newborns that may benefit from a targeted therapeutic approach is essential to improve future outcomes in neonatology. Presented here is the development and external validation of a multivariable diagnostic prediction rule for histological chorioamnionitis and histological chorioamnionitis with fetal involvement at birth in preterm newborns. The models are composed of small sets of clinical variables readily available in everyday practice and are therefore broadly applicable and simple to use. The models' diagnostic properties are encouraging and appeal for further evaluation to assess their value in supporting clinical decision-making.

## References

[1] Lahra MM, Jeffery HE (2004) A fetal response to chorioamnionitis is associated with early survival after preterm birth. Am J Obstet Gynecol 190: 147–151. 14749651 10.1016/j.ajog.2003.07.012

[2] Been JV, Rours IG, Kornelisse RF, Lima Passos V, Kramer BW, et al (2009) Histologic chorioamnionitis, fetal involvement, and antenatal steroids: effects on neonatal outcome in preterm infants. Am J Obstet Gynecol 201: 587 e581–588. 19729143 10.1016/j.ajog.2009.06.025

[3] Been JV, Zimmermann LJ (2009) Histological chorioamnionitis and respiratory outcome in preterm infants. Arch Dis Child Fetal Neonatal Ed 94: F218–225. 19131431 10.1136/adc.2008.150458

[4] Gantert M, Been JV, Gavilanes AW, Garnier Y, Zimmermann LJ, et al (2010) Chorioamnionitis: a multiorgan disease of the fetus? J Perinatol 30 Suppl: S21–30. 20877404 10.1038/jp.2010.96

[5] Lahra MM, Beeby PJ, Jeffery HE (2009) Maternal versus fetal inflammation and respiratory distress syndrome: a 10-year hospital cohort study. Arch Dis Child Fetal Neonatal Ed 94: F13–16. 18463119 10.1136/adc.2007.135889

[6] Elimian A, Verma U, Beneck D, Cipriano R, Visintainer P, et al (2000) Histologic chorioamnionitis, antenatal steroids, and perinatal outcomes. Obstet Gynecol 96: 333–336. 10960621 10.1016/s0029-7844(00)00928-5

[7] Meneses J, Bhandari V, Alves JG, Herrmann D (2011) Noninvasive ventilation for respiratory distress syndrome: a randomized controlled trial. Pediatrics 127: 300–307. 21262883 10.1542/peds.2010-0922

[8] Been JV, Rours IG, Kornelisse RF, Jonkers F, de Krijger RR, et al (2010) Chorioamnionitis alters the response to surfactant in preterm infants. J Pediatr 156: 10–15. 19833352 10.1016/j.jpeds.2009.07.044

[9] Watterberg KL, Gerdes JS, Cole CH, Aucott SW, Thilo EH, et al (2004) Prophylaxis of early adrenal insufficiency to prevent bronchopulmonary dysplasia: a multicenter trial. Pediatrics 114: 1649–1657. 15574629 10.1542/peds.2004-1159

[10] Lee HJ, Kim EK, Kim HS, Choi CW, Kim BI, et al (2010) Chorioamnionitis, respiratory distress syndrome and bronchopulmonary dysplasia in extremely low birth weight infants. J Perinatol 31: 166–170. 20724990 10.1038/jp.2010.113

[11] Inatomi T, Oue S, Ogihara T, Hira S, Hasegawa M, et al (2012) Antenatal exposure to Ureaplasma species exacerbates bronchopulmonary dysplasia synergistically with subsequent prolonged mechanical ventilation in preterm infants. Pediatr Res 71: 267–273. 22258085 10.1038/pr.2011.47

[12] Van Marter LJ, Dammann O, Allred EN, Leviton A, Pagano M, et al (2002) Chorioamnionitis, mechanical ventilation, and postnatal sepsis as modulators of chronic lung disease in preterm infants. J Pediatr 140: 171–176. 11865267 10.1067/mpd.2002.121381

[13] Lahra MM, Beeby PJ, Jeffery HE (2009) Intrauterine inflammation, neonatal sepsis, and chronic lung disease: a 13-year hospital cohort study. Pediatrics 123: 1314–1319. 19403497 10.1542/peds.2008-0656

[14] Redline RW, Faye-Petersen O, Heller D, Qureshi F, Savell V, et al (2003) Amniotic infection syndrome: nosology and reproducibility of placental reaction patterns. Pediatr Dev Pathol 6: 435–448. 14708737 10.1007/s10024-003-7070-y

[15] Tasci Y, Dilbaz B, Uzmez Onal B, Caliskan E, Dilbaz S, et al (2006) The value of cord blood interleukin-6 levels for predicting chorioamnionitis, funisitis and neonatal infection in term premature rupture of membranes. Eur J Obstet Gynecol Reprod Biol 128: 34–39. 16459014 10.1016/j.ejogrb.2005.11.049

[16] Yoon BH, Romero R, Park JS, Kim M, Oh SY, et al (2000) The relationship among inflammatory lesions of the umbilical cord (funisitis), umbilical cord plasma interleukin 6 concentration, amniotic fluid infection, and neonatal sepsis. Am J Obstet Gynecol 183: 1124–1129. 11084553 10.1067/mob.2000.109035

[17] Yoon BH, Romero R, Shim JY, Shim SS, Kim CJ, et al (2003) C-reactive protein in umbilical cord blood: a simple and widely available clinical method to assess the risk of amniotic fluid infection and funisitis. J Matern Fetal Neonatal Med 14: 85–90. 14629087 10.1080/jmf.14.2.85.90

[18] Zanardo V, Vedovato S, Cosmi E, Litta P, Cavallin F, et al (2010) Preterm premature rupture of membranes, chorioamnion inflammatory scores and neonatal respiratory outcome. BJOG 117: 94–98. 19781041 10.1111/j.1471-0528.2009.02358.x

[19] Been JV, Kornelisse RF, Rours IG, Passos VL, De Krijger RR, et al (2009) Early postnatal blood pressure in preterm infants: effects of chorioamnionitis and timing of antenatal steroids. Pediatr Res 66: 571–576. 19668111 10.1203/PDR.0b013e3181b7c4da

[20] Laupacis A, Sekar N, Stiell IG (1997) Clinical prediction rules. A review and suggested modifications of methodological standards. JAMA 277: 488–494. 9020274

[21] Toll DB, Janssen KJ, Vergouwe Y, Moons KG (2008) Validation, updating and impact of clinical prediction rules: a review. J Clin Epidemiol 61: 1085–1094. 19208371 10.1016/j.jclinepi.2008.04.008

[22] Adams ST, Leveson SH (2012) Clinical prediction rules. BMJ 344: d8312. 22250218 10.1136/bmj.d8312

[23] Lewis SH, Perrin E (1999). Pathology of the placenta, 2nd ed. New York: Churchill Livingstone. pp. 318–320.

[24] Langston C, Kaplan C, Macpherson T, Manci E, Peevy K, et al (1997) Practice guideline for examination of the placenta: developed by the Placental Pathology Practice Guideline Development Task Force of the College of American Pathologists. Arch Pathol Lab Med 121: 449–476. 9167599

[25] Naeye RL (1987) Functionally important disorders of the placenta, umbilical cord, and fetal membranes. Hum Pathol 18: 680–691. 3297994 10.1016/s0046-8177(87)80239-3

[26] Bendon RW, Faye-Petersen O, Pavlova Z, Qureshi F, Elder N, et al (1997) Histologic features of chorioamnion membrane rupture: development of methodology. Pediatr Pathol Lab Med 17: 27–42. 9050058

[27] Aina-Mumuney AJ, Althaus JE, Henderson JL, Blakemore MC, Johnson EA, et al (2007) Intrapartum electronic fetal monitoring and the identification of systemic fetal inflammation. J Reprod Med 52: 762–768. 17939590

[28] van de Laar R, van der Ham DP, Oei SG, Willekes C, Weiner CP, et al (2009) Accuracy of C-reactive protein determination in predicting chorioamnionitis and neonatal infection in pregnant women with premature rupture of membranes: a systematic review. Eur J Obstet Gynecol Reprod Biol 147: 124–129. 19819609 10.1016/j.ejogrb.2009.09.017

[29] McElrath TF, Hecht JL, Dammann O, Boggess K, Onderdonk A, et al (2008) Pregnancy disorders that lead to delivery before the 28th week of gestation: an epidemiologic approach to classification. Am J Epidemiol 168: 980–989. 18756014 10.1093/aje/kwn202 PMC2720771

[30] Kayem G, Maillard F, Schmitz T, Jarreau PH, Cabrol D, et al (2009) Prediction of clinical infection in women with preterm labour with intact membranes: a score based on ultrasonographic, clinical and biological markers. Eur J Obstet Gynecol Reprod Biol 145: 36–40. 19406555 10.1016/j.ejogrb.2009.03.023

[31] Jung HJ, Park KH, Kim SN, Hong JS, Oh KJ, et al (2011) Non-invasive prediction of intra-amniotic inflammation in women with preterm labor. Ultrasound Obstet Gynecol 37: 82–87. 21031346 10.1002/uog.8869

[32] Palacio M, Cobo T, Bosch J, Filella X, Navarro-Sastre A, et al (2009) Cervical length and gestational age at admission as predictors of intra-amniotic inflammation in preterm labor with intact membranes. Ultrasound Obstet Gynecol 34: 441–447. 19731395 10.1002/uog.6437

[33] Balaguer A, Alvarez-Serra J, Iriondo M, Gomez-Roig MD, Krauel X (2011) Rethinking classification of prematurity: a new clinical algorithm that improves etiologic assignment of preterm births. Neonatology 99: 295–301. 21135565 10.1159/000320153

[34] Miyano A, Miyamichi T, Nakayama M, Kitajima H, Shimizu A (1998) Differences among acute, subacute, and chronic chorioamnionitis based on levels of inflammation-associated proteins in cord blood. Pediatr Dev Pathol 1: 513–521. 9724338 10.1007/s100249900070

[35] Naccasha N, Hinson R, Montag A, Ismail M, Bentz L, et al (2001) Association between funisitis and elevated interleukin-6 in cord blood. Obstet Gynecol 97: 220–224. 11165585 10.1016/s0029-7844(00)01149-2

[36] Lee JC, Ahern TP, Chaves FP, Quillen K (2010) Utility of hematologic and volume, conductivity, and scatter parameters from umbilical cord blood in predicting chorioamnionitis. Int J Lab Hematol 32: 351–359. 19793112 10.1111/j.1751-553X.2009.01187.x

[37] Kidokoro K, Furuhashi M, Kuno N, Ishikawa K (2006) Amniotic fluid neutrophil elastase and lactate dehydrogenase: association with histologic chorioamnionitis. Acta Obstet Gynecol Scand 85: 669–674. 16752257 10.1080/01443610600604432

[38] Buhimschi CS, Bhandari V, Hamar BD, Bahtiyar MO, Zhao G, et al (2007) Proteomic profiling of the amniotic fluid to detect inflammation, infection, and neonatal sepsis. PLoS Med 4: e18. 17227133 10.1371/journal.pmed.0040018 PMC1769412

[39] Holst RM, Hagberg H, Wennerholm UB, Skogstrand K, Thorsen P, et al (2011) Prediction of microbial invasion of the amniotic cavity in women with preterm labour: analysis of multiple proteins in amniotic and cervical fluids. BJOG 118: 240–249. 21054762 10.1111/j.1471-0528.2010.02765.x

[40] Cotten CM, Taylor S, Stoll B, Goldberg RN, Hansen NI, et al (2009) Prolonged duration of initial empirical antibiotic treatment is associated with increased rates of necrotizing enterocolitis and death for extremely low birth weight infants. Pediatrics 123: 58–66. 19117861 10.1542/peds.2007-3423 PMC2760222

[41] Clark RH, Bloom BT, Spitzer AR, Gerstmann DR (2006) Empiric use of ampicillin and cefotaxime, compared with ampicillin and gentamicin, for neonates at risk for sepsis is associated with an increased risk of neonatal death. Pediatrics 117: 67–74. 16396862 10.1542/peds.2005-0179

[42] Kuppala VS, Meinzen-Derr J, Morrow AL, Schibler KR (2011) Prolonged initial empirical antibiotic treatment is associated with adverse outcomes in premature infants. J Pediatr 159: 720–725. 21784435 10.1016/j.jpeds.2011.05.033 PMC3193552

